# Pre-Exposure Prophylaxis Adherence and HIV Self-Testing App Among Women in the South Bronx: 12-Month Usability, Acceptability, and Feasibility Study

**DOI:** 10.2196/86407

**Published:** 2026-06-02

**Authors:** Connor G Wright, Terry L Chern, Tara F Abularrage, Julie Franks, Ann Kahn, Shakir Edwards, Harshit H Chellani, Rashaunna Redd, Alexander W Ying, Siddarth Arumugam, Robert Stanciu, Samuel K Sia, Ellen AB Morrison, Jessica E Justman

**Affiliations:** 1 ICAP at Columbia University, 722 W 168th Street, New York, NY, 10032, United States, 1 212 342 0505; 2 School of Engineering and Applied Science, Columbia University, New York, NY, United States

**Keywords:** HIV prevention, PrEP adherence, pre-exposure prophylaxis, digital health, women, HIV self-testing, risk perception

## Abstract

**Background:**

HIV pre-exposure prophylaxis (PrEP) is underused by cis- and transgender women despite a significant HIV burden. Smartphone technologies are promising tools to support HIV prevention but have yet to be assessed in women.

**Objective:**

We conducted a 12-month feasibility study to assess the use and acceptability of a mobile phone app, SmartPrEP, designed to support PrEP adherence and HIV self- and partner-testing among women living in an area of elevated HIV burden in New York City.

**Methods:**

Nonpregnant adult cisgender and transgender women who met US PrEP eligibility criteria and were PrEP naïve, reported PrEP use for <3 months, or had inconsistent PrEP use were eligible. Participants received oral PrEP and HIV self-testing kits and downloaded the SmartPrEP app, which sent daily reminders to take PrEP and record adherence through the app. PrEP adherence was assessed based on participants’ self-recorded average doses per week as entered in the app. Sexual behaviors and app acceptability were evaluated quarterly by interviewer-administered questionnaires.

**Results:**

From February 2022 to August 2023, 40 participants were enrolled in the study. Median age was 30 (IQR 24-35) years, 70% (28/40) identified as cisgender women, 30% (12/40) as transgender women, 48% (19/40) as Hispanic, and 35% (14/40) as Black. At baseline, the majority, 80% (32/40), had no history of PrEP use, and 65% (26/40) reported that they did not believe they were at risk of HIV. However, 90% (36/40) reported ≥1 and 25% (10/40) reported >4 HIV risk behaviors in the past 6 months, with 58% (23/40) reporting anal or vaginal sex with more than 1 partner. Over the course of the study, although 8 participants withdrew early, and 14 were lost to follow-up, there were 2 pregnancies and 1 HIV seroconversion. PrEP adherence was low, with 80% (32/40) recording <3 doses per week, 17% (7/40) recording 3‐5 doses, and 3% (1/40) recording ≥6 doses per week. PrEP adherence averaged over the second half of study participation was lower than adherence in the first half, with only 10% (4/40) recording >3 doses per week compared to 20% (8/40). In total, 4 participants conducted HIV self- or partner-testing using SmartPrEP during study follow-up. App acceptability assessed at month 12 was moderate to high (median score 3.71 of max 5, IQR 3.47‐4.16).

**Conclusions:**

Despite consistently rating the app as acceptable and receiving quarterly HIV testing and counseling, most participants did not achieve optimal PrEP adherence, demonstrating the limitations of this mobile health app among women at elevated risk of HIV. Active navigation and strategies to address gaps in risk perception among women will remain critical, as new, long-acting formulations of PrEP become available.

## Introduction

Women continue to bear a significant burden of the HIV epidemic in the United States, accounting for 20% of all diagnoses in 2022 [1]. Longstanding and interlocking individual, systemic, and structural factors have driven the racial, ethnic, and economic disparities that mark the US HIV epidemic [2]. Black women, who comprise 13% of the US female population, represented 50% of 2022 HIV diagnoses among women [1]. Transgender people, who comprise an estimated 0.6% of the US population aged 13 years and older, are also disproportionately vulnerable to HIV, accounting for 2% of 2022 diagnoses [1,3]. Within the latter group, 87% were transgender women, highlighting their uniquely severe burden of HIV. The 2019 US Strategic Plan to End the HIV Epidemic prioritizes effective prevention interventions for Black women and transgender women [4,5].

Pre-exposure prophylaxis (PrEP) in oral and long-acting injectable formulations is effective as HIV prevention, but disparities in use undermine its potential to slow the US epidemic [5,6]. Only 8% of 2023 PrEP users were women, despite women accounting for nearly 20% of 2022 diagnoses. Black and Latinx individuals represented 14% and 17% of PrEP users, and 39% and 31% of HIV diagnoses, respectively [1,7]. Barriers to PrEP uptake and use among women include limited discussion of HIV prevention and PrEP in women-focused primary and sexual health care settings, limited competency in PrEP counseling and services among health care providers who provide care to women at risk, and a dearth of public health campaigns and social media messaging tailored to women [8]. These gaps, in turn, perpetuate persistent stigma around discussions of HIV risk, prevention, and care; low self-perceived HIV risk; and low awareness of and trust in PrEP as an effective HIV prevention strategy among communities of women at elevated risk of HIV acquisition [9-12]. While transgender women have a relatively high awareness of PrEP [13], they also experience heightened barriers to its use, including structural barriers such as unstable housing and lack of access to health care, pill fatigue related to daily oral PrEP, and concerns about possible drug-drug interactions affecting gender-affirming hormone therapy [14-16].

Guidelines recommend offering PrEP as part of a comprehensive approach to HIV prevention, including regular testing to know one’s HIV status and the status of one’s sexual partners. HIV self-testing (HIVST) involves individuals collecting their own blood or oral fluid, performing a rapid HIV test, and interpreting the result in a private setting, either alone or with others [17,18], facilitating awareness of one’s status and that of one’s partners. HIVST thus can play a supporting and complementary role in comprehensive HIV prevention. HIVST has been incorporated into PrEP programs in other parts of the world [19], and HIVST-supported PrEP delivery models have demonstrated acceptability and feasibility among diverse populations, with similar adherence and retention outcomes compared to standard-of-care PrEP delivery [19]. Current US PrEP guidelines support HIVST as an additional strategy to increase testing for those at risk, but they do not endorse oral fluid-based HIVST to determine HIV status in clients initiating or continuing PrEP [20]. While conventional HIV testing is widely available without cost in the United States, HIVST is available through limited not-for-profit settings and commercial retailers with prices of approximately US $45 per test as of 2026, contributing to its underuse among populations at risk of HIV acquisition in the United States [21].

Digital health interventions, including mobile health (mHealth) tools, have the potential to facilitate a wide range of HIV prevention interventions in nonclinical settings, including supporting PrEP adherence, HIVST, and remote data collection for prevention research [22,23]. One such intervention, the “SMARTtest” app, was developed to facilitate the use and interpretation of results of a dual blood-based HIV or syphilis rapid test [24]. Evaluated among cisgender men and transgender women who have sex with men in New York City, the app demonstrated high acceptability and high ratings for functionality and helpfulness, suggesting that it could increase the frequency of self- and partner-testing [25,26]. While studies show that mHealth interventions are feasible, acceptable, and potentially effective means of addressing the HIV prevention needs of gay, bisexual, and other men who have sex with men and transgender women, few have been designed to engage both cis- and transgender women [27,28]. Emerging evidence suggests that mHealth tools developed for women demonstrate high acceptability, but further research is needed to assess efficacy and potential impact on PrEP adherence [29,30]. HIVST with digital support has also been shown to be feasible, acceptable, and preferable, particularly among first-time testers and hard-to-reach populations, including transgender women [23,31]. Additionally, app-based data collection has been viewed as empowering, offering enhanced privacy, confidentiality, and a greater sense of safety for transgender women participating in HIV prevention research [23]. In this study, the SMARTtest app was adapted to create “SmartPrEP”—a mobile app designed to support PrEP adherence and HIV self- and partner-testing among cis- and transgender women.

New York City is a long-standing epicenter of the US HIV epidemic, with HIV diagnoses concentrated in neighborhoods with overlapping systemic and structural disparities that heighten vulnerability [1,32,33]. Since at least 2019, the Bronx has consistently experienced the highest rate per 100,000 of new HIV diagnoses as compared to other New York City boroughs, with 372 individuals newly diagnosed in 2023 [32,33]. HIV prevalence in the Bronx was 2% in 2022, comparable to countries in Africa such as Nigeria (1.3%), Côte d’Ivoire (2.9%), and Kenya (3.7%) [34-36]. In 2022, among all counties in the United States, the Bronx had the greatest number (n=9454) and highest case rate (1552 per 100,000) of women living with an HIV diagnosis [37].

The purpose of this study was to assess a novel smartphone app designed to provide PrEP adherence reminders and facilitate HIVST to support HIV prevention for women at elevated risk of HIV. We report here patterns of self-reported PrEP adherence and HIV self- and partner-testing as collected through the SmarPrEP app and app acceptability among sexually active, HIV-negative cis- and transgender women living in the South Bronx.

## Methods

### Study Design and Eligibility Criteria

We conducted a 12-month, single-arm, open-label, feasibility study among a sample of cis- and transgender women to assess the acceptability and frequency of use of a smartphone app designed to support HIV prevention. Nonpregnant adults aged 18 years or older who were willing and able to provide informed consent; self-identified as cis- or transgender women; were eligible for PrEP based on the 2021 US Centers for Disease Control and Prevention (CDC) criteria [6]; and were either not currently taking PrEP, or reported PrEP use for <3 months, or had inconsistent PrEP use of any duration were eligible to participate in the study.

### Study Procedures

At enrollment, participants received oral PrEP medication and adherence counseling as well as 3 HIVST kits (OraQuick oral fluid self-test kit, OraSure Technologies, Inc). Study staff assisted participants in downloading the “SmartPrEP” smartphone app, demonstrated how to personalize the daily PrEP adherence reminders, and provided HIVST and partner testing support. In keeping with national guidelines at the time of the study, transgender women could choose to receive either Truvada (tenofovir disoproxil fumarate [TDF] 300 mg/emtricitabine [FTC] 200 mg) daily or Descovy (tenofovir alafenamide 25 mg/FTC 200 mg); cisgender women received Truvada (TDF 300 mg/FTC 200 mg). Participants were classified as in track A if they received their oral PrEP medication and associated testing at the Bronx study site or track B if this was provided by an external medical provider. In both tracks, participants received a combination HIV prevention intervention that included PrEP prescription provided directly at the Bronx study site (track A) or provided by an external medical provider (track B); regular PrEP follow-up services, including HIV testing, pregnancy, and bacterial sexually transmitted infection (STI) testing (syphilis, gonorrhea, and chlamydia); and additional free HIVST kits at each study visit that could be used by participants or their partners as desired. All participants received adherence counseling, HIV or STI counseling, condoms, and lubricant. Cisgender women also received family planning counseling. Participant compensation is described below.

### SmartPrEP App Overview

The development of the SmartPrEP app (Multimedia Appendix 1) was based largely on the previously developed SMARTtest app, which was created through a user-informed app development process with men who have sex with men and transgender women in New York City [24]. The SMARTtest app showed positive user feedback and high ratings for functionality and helpfulness, including impressions that the app was easy to use and convenient [25,26]. The subsequently developed SmartPrEP app used in this study was built with the ReactNative framework and allowed for simultaneous cross-platform development of both an iOS and Android app. This ensured that the app was compatible with Android versions 7.0 or later smartphones and with Apple iOS smartphones, including subsidized devices and service plans available through the federal Lifeline program [38]. Core SmartPrEP app features included (1) private user account creation; (2) customization of push notification PrEP reminders, which included notification title and body text and 2 distinct daily notification timings; (3) a calendar feature to track PrEP use; (4) detailed instructions on how to perform a rapid OraQuick HIV test on oneself or partners; (5) objective and automated test scanning and result logging; (6) features to share, delete, or save test results; and (7) information on additional HIV prevention resources and nearby clinics, including additional HIV testing locations (Multimedia Appendix 2). For the algorithmic and automated test scanning and result logging feature, a cloud-based machine-learning inference algorithm was used to interpret test results and enable self-test logging. This algorithm used custom-developed image recognition and processing techniques tailored to the classification of HIV rapid test images, related similarly to techniques published previously [39].

### Participant Recruitment

We recruited participants by direct internet and printed advertisements in high HIV prevalence zip codes of the Bronx and Manhattan. Local PrEP providers were informed of the study and encouraged to refer eligible patients. The study was discussed with the research site’s community advisory board, and advertisements were posted on social media. Study staff also reached out to local women-led and lesbian, gay, bisexual, transgender, and queer or questioning organizations for potential participant referral and advertising. Enrolled participants were encouraged to refer potentially eligible friends and received a modest amount of additional compensation (US $30) if their referral resulted in an enrollment.

### Study Setting

Study procedures took place at a clinical research site located in a South Bronx storefront easily accessible by public transport. The study design and setting intentionally approximated routine service delivery conditions. Participants were provided PrEP medication; HIV, STI, and safety laboratory testing according to the 2021 guidelines [6]; and HIVST kits at no cost; and study staff were available to answer sexual health-related questions.

### Data Collection

Participants completed 6 study visits during the 12-month follow-up period. Information on baseline demographics, sexual behavior, and mobile app use was collected at the enrollment visit via an interviewer-administered electronic questionnaire.

The question “Do you personally believe you are at risk of getting HIV?” assessed HIV risk perception at baseline and at quarterly visits. Sexual behavior questionnaires used PrEP eligibility questions sourced from the CDC’s 2021 national guidelines, and SmartPrEP acceptability questionnaires were informed by the SMARTtest study [6,25]. A brief month 1 visit was conducted to provide results of screening tests to participants, HIV or STI prevention counseling, and the first follow-up acceptability assessment about their use of the SmartPrEP app. Participants then completed 4 quarterly in-person follow-up visits, which included follow-up assessments of sexual behavioral risk, acceptability of the SmartPrEP app, satisfaction with the app, and experiences with self- and partner-testing via an interviewer-administered questionnaire. In May 2022, more detailed questions on PrEP and HIVST history use were added to the baseline questionnaire, and the related data were available for a subset of participants (n=29) based on enrollment dates.

Separately from the in-person visits, the SmartPrEP app facilitated daily remote data collection. This included data on daily logged PrEP doses (PrEP doses could be backlogged up to 3 days), reasons for missing a PrEP dose, results of any HIVST conducted by the participant and/or partners and results of the test if the partner gave consent for results to be saved, and reasons for conducting self- or partner-testing. Data logged in the app were uploaded instantaneously for analysis.

### Statistical Analysis

Descriptive analyses of demographic, behavioral, and app acceptability data collected from interviewer-administered questionnaires at each study visit were conducted using SAS (version 9.4; SAS Institute Inc). To assess bias due to attrition, we used the Fisher exact test to compare demographics, sexual behaviors, and history of PrEP use among those who completed 12 months of follow-up and those who withdrew or were lost to follow-up.

In total, 19 questions assessing SmartPrEP acceptability were scored on a 5-point Likert scale (1=strongly disagree to 5=strongly agree) and were assessed at months 3, 6, and 12. Questions on acceptability were grouped into 5 thematic areas: app usability and engagement, behavioral impact (PrEP and HIVST), relevance and trust, knowledge and awareness, and partner communication and testing. PrEP adherence was assessed using daily self-reported data on PrEP doses from the SmartPrEP app, and average weekly adherence was calculated for each participant. To characterize the relationship between PrEP adherence, SmartPrEP acceptability, and sexual behaviors, we conducted stratified analyses of data collected at months 3, 6, and 12.

To visualize patterns of PrEP adherence and engagement in sexual behaviors, a heat map was generated in Python (version 3.8.5; Python Software Foundation). Each participant’s weekly self-reported PrEP adherence was displayed temporally alongside their retrospective self-reported sexual behaviors collected quarterly from enrollment to month 12. Weekly PrEP adherence was calculated by participants’ PrEP use log in SmartPrEP as the number of “yes” responses to daily dosing queries per week (1‐7 doses per week). Participant sexual behaviors were scored quarterly based on self-reported engagement during the prior 3 months in 9 behaviors associated with HIV acquisition (1 for “yes,” 0.5 for “do not know,” and 0 for “no”). Data on participant study enrollment date and date of study completion, withdrawal, or loss to follow-up were aggregated and harmonized to create comparable follow-up periods (aligned to the starting week for each individual). For participants who were lost to follow-up or withdrew from the study, an intention-to-treat approach was conducted, and PrEP adherence was assessed for the full 12 months of the study duration, unless otherwise stated.

### Ethical Considerations

This study [AAAT7092] was reviewed and approved by the Columbia University Irving Medical Center Institutional Review Board. All participants provided written consent to participate in the study, including a formal screening visit. To maintain participant confidentiality, all study data were stored without personal identifiers and instead used a coded number. Records containing personal identifiers were stored separately and did not contain the coded number. A link log that linked participant names with the coded numbers did not include personal health information and was maintained in a separate location. Participants were moderately compensated for their participation in this study (US $50‐$100 per visit; US $475 total for full completion) and a round-trip metro card for travel to the study site. Additional compensation was available for those who referred participants who later enrolled in the study (US $30).

## Results

Between January 2022 and August 2023, 104 individuals were prescreened, with 52 completing a full screening visit at the research site. Among those screened as eligible, 40 participants consented to be enrolled in the study, with 36 in track A (PrEP received at the Bronx site) and 4 in track B (PrEP received from an external provider).

### Baseline Demographics, Health, and General App Use

The median age of participants was 30 (IQR 24-35) years, with 70% (28/40) identifying as cisgender women and 30% (12/40) as transgender women (Table 1). In total, 43% (17/40) of the women identified as Black, 48% (19/40) as Hispanic, and 18% (7/40) as White. Most participants were not in a relationship (28/40, 70%) and were not employed (23/40, 58%). Educational attainment was heterogeneous, with 58% (23/40) completing at least secondary school and 23% (9/40) completing college or university or higher. A third of participants (13/40, 33%) reported not having a primary care physician, and 53% (21/40) reported a prior diagnosis of anxiety, depression, bipolar disorder, schizophrenia, or other psychiatric illness (Table 1).

**Table 1.** Participant demographics and general app acceptability at baseline (N=40).

Demographics	Values
Characteristic	
Age (years), median (IQR)	30 (24-35)
Gender identity, n (%)	
Cisgenderwoman	28 (70)
Transgender woman	12 (30)
Relationship status, n (%)	
Married or civil union or legal partnership	3 (8)
In a relationship	9 (23)
Single or divorced or widowed	28 (70)
Employment status, n (%)	
Full-time employment	11 (28)
Part-time employment	6 (15)
Not employed	23 (58)
Education, n (%)	
Completed primary school	8 (20)
Completed secondary school	12 (30)
Completed technical school	2 (5)
Some college or university or higher	9 (23)
Completed college or university or higher	9 (23)
Race and ethnicity, n (%)	
African American, Afro-Caribbean, or Black	14 (35)
Filipino	1 (3)
Hispanic	19 (48)
White	4 (10)
Multiracial	2 (5)
Ever been diagnosed with anxiety, depression, bipolar disorder, schizophrenia, or any other psychiatric illness? n (%)	
Yes	21 (53)
No	19 (48)
Have a primary care MD^a^? n (%)	
Yes	27 (68)
No	13 (33)
General app acceptability	
How often do you use mobile apps on your phone (not including texting and calling)? n (%)	
Every day	37 (93)
Most days	2 (5)
Rarely	1 (3)
How easy is it for you to download a new mobile app? n (%)	
Very easy	31 (78)
Easy	4 (10)
Neither easy nor difficult	2 (5)
Difficult	3 (8)
Very difficult	0 (0)
How easy is it for you to understand and use a newly downloaded mobile app? n (%)	
Very easy	25 (63)
Easy	11 (28)
Neither easy nor difficult	3 (8)
Difficult	1 (3)
Very difficult	0 (0)
When using a newly downloaded mobile app, how easy is it for you to change the app’s notification features on your phone? n (%)	
Very easy	22 (55)
Easy	11 (28)
Neither easy nor difficult	5 (13)
Difficult	2 (5)
Very difficult	0 (0)
How likely are you to download a new mobile app that supports your overall health? n (%)	
Very likely	19 (48)
Likely	13 (33)
Neither likely nor unlikely	4 (10)
Unlikely	2 (5)
Very unlikely	2 (5)

a MD: medical doctor.

Participants were asked about HIV risk in the 6 months prior to enrollment (Table 2). In total, 35% (14/40) of participants believed that they were at risk of getting HIV, while 58% (23/40) believed that they were not, and 8% (3/40) reported they “do not know.” A total of 22 (55%) participants reported ever having had a bacterial STI, and 8 (20%) participants had been diagnosed with an STI in the last 6 months. Regarding sexual behaviors, 58% (23/40) of participants reported anal or vaginal sex with more than 1 partner, and 25% (10/40) reported having sex with male partners who may have other male partners. Over one-third of participants, 38% (15/40), reported having engaged in condomless sex with someone whose HIV status was unknown, and 5% (2/40) had sex with a person living with HIV with a known unsuppressed viral load. Transactional sex was reported by 20% (8/40) of participants, and 20% (8/40) had partners involved in transactional sex. Overall, 90% (36/40) of participants reported ≥1 behavior indicated above that could increase potential exposure to HIV, and 25% (10/40) reported >4 such behaviors in the prior 6 months.

**Table 2.** Baseline behaviors and perception associated with sexual health.

Question	Values, n (%)
Do you personally believe you are at risk of getting HIV?	
Yes	14 (35)
No	23 (58)
Do not know	3 (8)
In the last 6 months, have you taken medications because of a possible exposure to HIV?	
Yes	4 (10)
No	36 (90)
In the last 6 months, have you been diagnosed with a sexually transmitted infection?	
Yes	8 (20)
No	31 (78)
Do not know	1 (3)
In the last 6 months, have you had anal or vaginal sex with more than 1 sexual partner?	
Yes	23 (58)
No	15 (38)
Do not know	2 (5)
In the last 6 months, have you had anal or vaginal sex with a male partner who may have sexual partners who identify as cisgender male individuals or were assigned male at birth?	
Yes	10 (25)
No	21 (53)
Do not know	9 (23)
In the last 6 months, have you had anal or vaginal sex without using a condom with a person whose HIV status was unknown?	
Yes	15 (38)
No	20 (50)
Do not know	5 (13)
In the last 6 months, have you engaged in anal or vaginal sex without using a condom with a person who is HIV positive and has an unsuppressed viral load?	
Yes	2 (5)
No	32 (80)
Do not know	6 (15)
In the last 6 months, have you been involved in transactional sex (ie, sex for money, drugs, food, or housing), including commercial sex work?	
Yes	8 (20)
No	30 (75)
Do not know	2 (5)
In the last 6 months, have you had sex with partners who may be involved in transactional sex (ie, sex for money, drugs, food, or housing), including commercial sex work?	
Yes	8 (20)
No	22 (55)
Do not know	10 (25)
Have you ever used pre-exposure prophylaxis (PrEP)?	
Yes	8 (20)
No	32 (80)
Have you taken PrEP within the last 6 months?	
Yes	7 (88)
No	1 (13)
Within the past month, on how many days did you take PrEP pills?	
30 days	3 (43)
29 days	2 (29)
Less than 10 days	2 (29)
Have you ever heard of PrEP before hearing about this study?^a^	
Yes	20 (69)
No	9 (31)
When was your most recent HIV test?^a^	
3 months or less	24 (83)
3‐12 months	4 (14)
More than 1 year	1 (4)
Have you ever used an in-home HIV test kit to test yourself (eg, OraQuick)?^a^	
Yes	2 (7)
No	27 (93)
Have you ever used an in-home HIV test kit to test a sexual partner?^a^	
Yes	1 (4)
No	28 (97)

a Question only available for a subset of the study sample (n=29).

STI testing at enrollment revealed 3 participants with serologic evidence of syphilis; all 3 were asymptomatic and reported a prior history of syphilis treatment. One of the 3 was also positive via nucleic acid amplification test (NAAT+) for chlamydia at enrollment. A fourth participant was positive via nucleic acid amplification test for chlamydia at month 6.

Among the 40 participants enrolled, the majority, 80% (32/40), had no history of any PrEP use; among the 8 who reported prior PrEP, 7 reported use in the past 6 months, and 3 reported daily adherence in the past month (Table 2). In total, 10% (4/40) reported taking medications after a possible HIV exposure in the last 6 months (eg, postexposure prophylaxis). A total of 29 participants who enrolled after May 2022 completed the baseline PrEP and HIVST history questions added during ongoing participant follow-up. Among those respondents, 69% (20/29) indicated that they had heard of PrEP before participating in this study (Table 2). Of the 29, 24 (83%) participants had received an HIV test in the past 3 months, with the most frequently reported reasons being “I get tested for HIV regularly” (10/29, 35%), followed by “It was part of my routine checkup” (8/29, 28%). In total, 2 (7%) participants reported having ever used an in-home HIVST kit. Among those who had not, 72% (21/29) reported it was because they were unaware of the self-testing option. Only 1 (4%) participant reported having ever used an in-home HIV test kit to test a sexual partner.

In assessing general mobile app use at baseline, most respondents (37/40, 93%) reported using mobile apps daily, with 90% (36/40) finding new apps very easy or easy to understand and use. Additionally, 80% (32/40) reported being very likely or likely to download a health-related app, and 55% (22/40) reported they found it very easy to modify a newly downloaded app’s notification features on their phone (Table 1).

### Follow-Up

**Figure 1.**
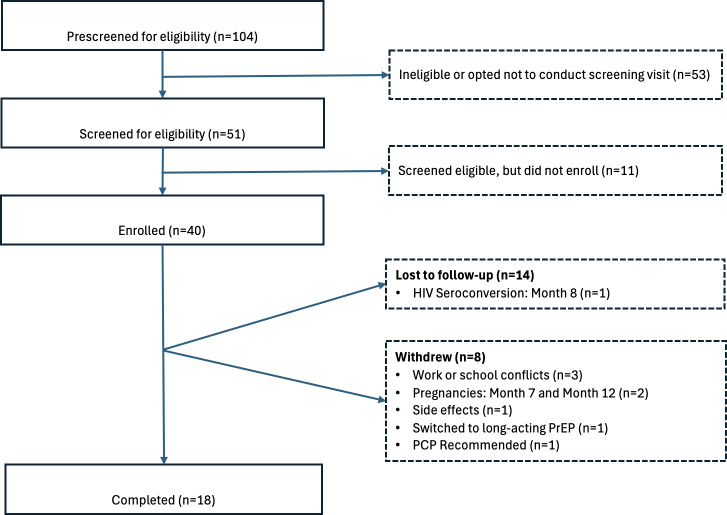
CONSORT flow diagram detailing participant prescreening, screening, enrollment, and retention. CONSORT: Consolidated Standards of Reporting Trials; PCP: primary care provider; PrEP: pre-exposure prophylaxis.

Of the 40 enrolled participants, 18 (45%) completed the full 12 months of follow-up (Figure 1). Compared to those who completed all follow-up visits, those who withdrew or were not retained were significantly more likely to be single, divorced, or widowed. Among those who completed the additional baseline PrEP and HIVST questions (n=29), three characteristics were associated with being more likely to complete the study: (1) taking medications in the 6 months before enrollment due to a possible HIV exposure, (2) having heard of PrEP prior to the study, and (3) having tested for HIV in the 3 months prior to enrollment (data not shown). Stratified analyses of the full sample evaluating differences between cisgender and transgender women were not found to be significant, and the 2 subpopulations were therefore grouped together.

More than half of participants continued to report that they believed they were not at risk of getting HIV over the course of the study (baseline: 23/40, 58%; month 6: 15/22, 68%; and month 12: 11/18, 61%).

During study follow-up, 1 participant became pregnant at month 7, and 1 participant had an HIV seroconversion at month 8; both participants exited the study early. An additional participant became pregnant during the study and had an uncomplicated, full-term delivery after completing the full 12 months of follow-up; this participant was 1 of the 3 with serologic evidence of syphilis at enrollment.

### At Home HIV Self- and Partner-Testing via SmartPrEP

In total, 4 of 40 participants conducted an at-home HIV test (self- or partner-testing) using the SmartPrEP app. Participants could select multiple reasons for conducting HIVST, and the 2 most common reasons were concern about partners’ sexual behavior and wanting to know their own HIV status. Among these participants, only 1 logged a dual partner HIVST. The highest frequency of HIVST use per participant in 12 months of follow-up was 3 times.

### Self-Reported PrEP Adherence via SmartPrEP

**Figure 2.**
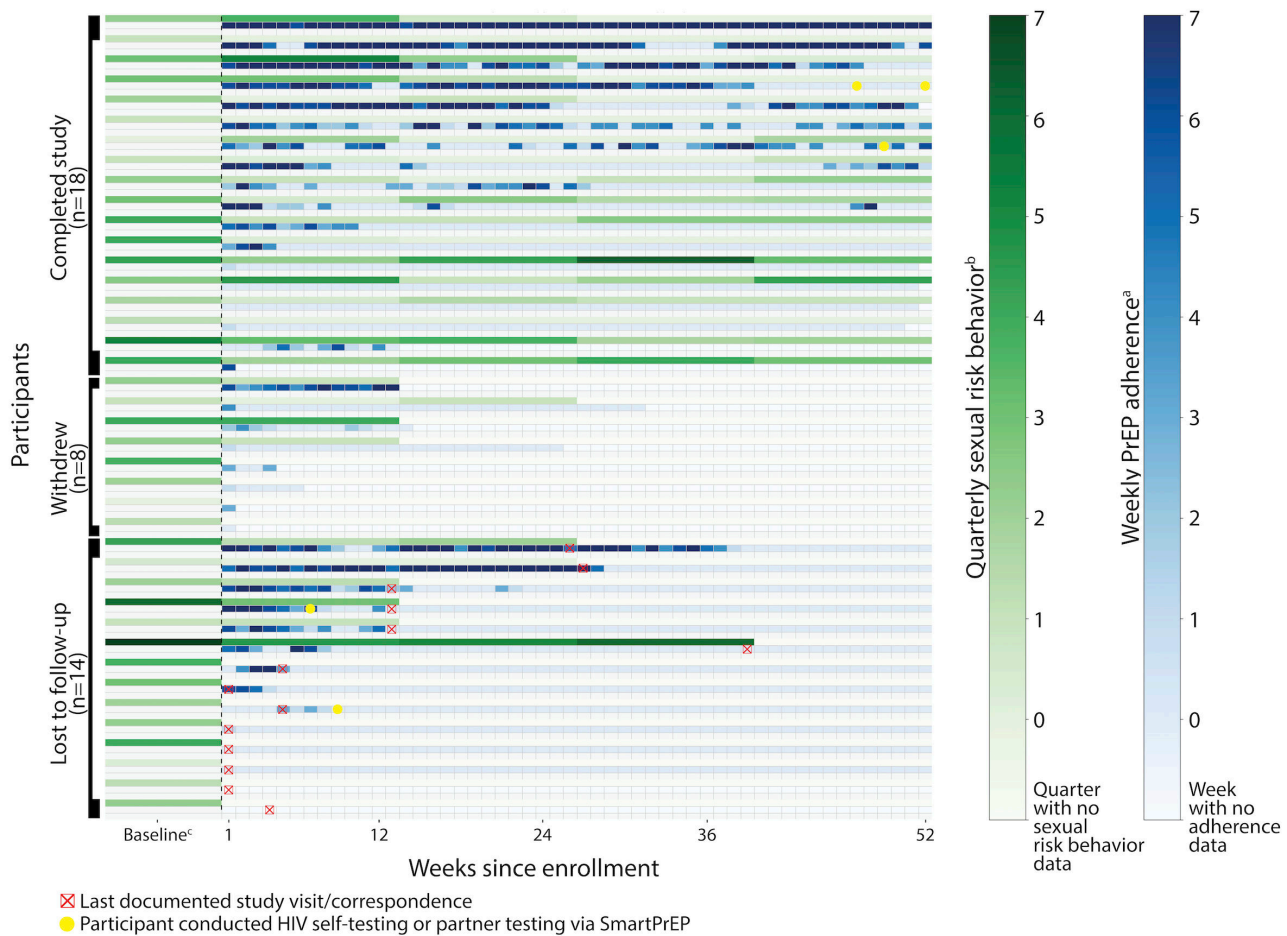
Heat map of weekly PrEP adherence and quarterly sexual risk behavior (N=40). PrEP: pre-exposure prophylaxis. ^a^Number of “yes” responses to daily dosing query from the app per week. ^b^Scored (summed) 9-question sexual risk behavior questionnaire (1=yes, 0.5=do not know, 0=no) per quarter. ^c^Baseline sexual risk behavior denotes sexual risk behavior for the past 6 months, recorded at baseline.

Weekly PrEP adherence as recorded by participants via the SmartPrEP app was heterogeneous (Figure 2). Through 12 months of follow-up, 32 of 40 (80%) participants self-reported an average of 0‐3 PrEP pills per week, 5 of 40 reported an average of 3‐5 weekly PrEP pills, and only 3 of 40 participants reported close to optimal adherence with an average of 5‐7 weekly PrEP pills. PrEP adherence declined between the first and last 26 weeks of follow-up: in the first 26 weeks, 7 (18%) participants reported optimal PrEP adherence, with an average of 5‐7 self-reported daily PrEP pills per week, compared to only 2 of 40 (5%) participants who maintained this level of adherence in the last 26 weeks. In the last 26 weeks, 36 of 40 (90%) participants logged an average of 0‐3 daily PrEP pills per week via SmartPrEP. Weekly PrEP adherence and quarterly sexual behavior data did not appear to have a correlation based on data visualization via the heat map.

There were some technical issues related to the app that occurred during study follow-up, including 2 outage periods where all active participants could not record PrEP adherence for 3 and 6 days, respectively. In total, 2 participants also had significant issues accessing the app on their phone, including the PrEP log. Among a total of 14,600 potential PrEP log days (365 days per 40 participants), approximately 685 of 14,600 (3.9%) were affected by outages; however, these did not demonstrate a pattern (data are not shown).

### SmartPrEP Acceptability

**Figure 3.**
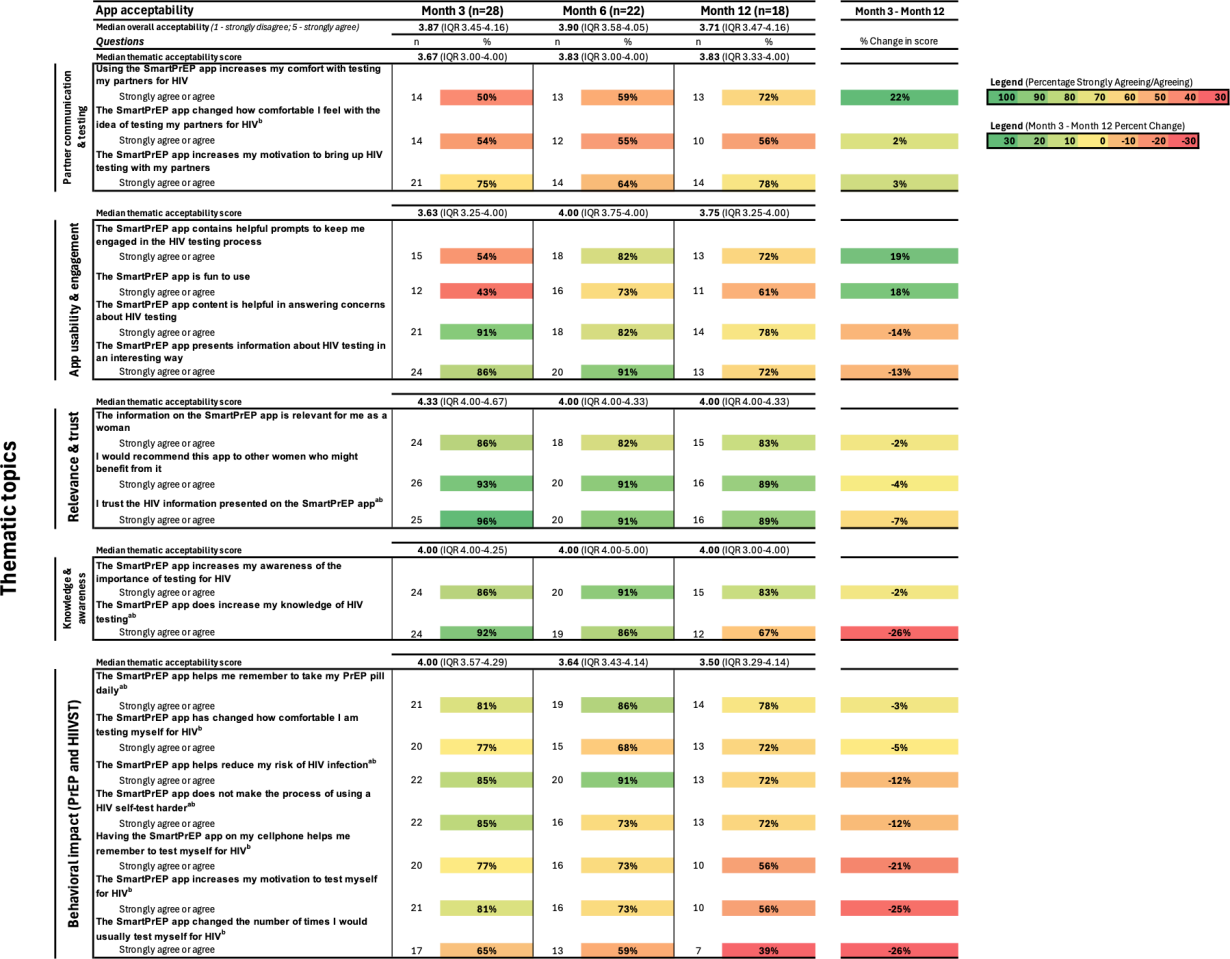
SmartPrEP app acceptability measures and thematic areas at months 3, 6, and 12. HIVST: HIV self-testing; PrEP: pre-exposure prophylaxis. ^a^Question was reverse-coded. ^b^Question includes missing data for month 3 (questions added to visit after n=2 participants had completed the visit).

After 3 months of follow-up, participants rated the SmartPrEP app moderately to highly acceptable, with a median overall acceptability score of 3.87 (IQR 3.45‐4.16; Figure 3). Among the thematic groups at month 3, the median score point estimate for relevance and trust was highest (median 4.33, IQR 4.00‐4.67), and usability and engagement was lowest (median 3.63, IQR 3.25‐4.00); however, these IQRs all overlapped. In total, 96% (25/26) agreed or strongly agreed that they trusted the HIV information presented on the SmartPrEP app, 93% (26/28) said that they would recommend this app to other women who might benefit from it, and 92% (24/26) said that the SmartPrEP app increased their knowledge of HIV testing. Participants were also interviewed about the behavioral impact of SmartPrEP: 85% (22/26) strongly agreed or agreed that the PrEP app helped reduce their risk of HIV infection, 85% (22/26) strongly agreed or agreed that the SmartPrEP app did not make the process of an HIV self-test harder, and 81% (21/26) strongly agreed or agreed that the app helped them remember to take their PrEP pill daily.

Among the 18 of 40 participants who completed 12 months of follow-up, median acceptability was 3.71 (IQR 3.47‐3.16; Figure 3). Participants reported high levels of perceived relevance and trust as well as knowledge and awareness of the SmartPrEP app (median 4.0, IQR 4.0‐4.33 and median 4.0, IQR 3.00‐4.00, respectively). Participants reported the highest levels of acceptability when asked, “I trust the HIV information presented on the SmartPrEP app” (16/18, 89%) and “I would recommend this app to other women who might benefit from it” (16/18, 89%).

Figure 3 also illustrates the percentage change in specific acceptability themes from month 3 to month 12. The greatest reported increases in acceptability from month 3 to month 12 included that the SmartPrEP app “increases my comfort with testing my partners for HIV” (+22%), “contains helpful prompts to keep me engaged in the HIV testing process” (+19%), and “is fun to use” (+19%). The largest decreases in acceptability were that the SmartPrEP app, “changed the number of times I would usually test myself for HIV” (−26%) and “increases my knowledge of HIV testing” (−26%).

### PrEP Adherence and Sexual Behavior and App Acceptability

Analysis of adherence by behavior (Multimedia Appendix 3) revealed that among enrolled participants who reported engaging in at least 1 sexual behavior associated with an elevated risk of HIV acquisition in the prior 3 months, 3 of 28 (18%) logged ≥4 doses of PrEP per week between baseline and month 3; adherence during this period of behavioral risk remained low, with only 5 of 22 (42%) logging >4 doses per week between month 3 and month 6, and 2 of 18 (22%) logging >4 doses per week between month 9 and month 12. Analysis of adherence by app acceptability (Multimedia Appendix 3) revealed that among participants who reported high app acceptability at month 3, only 1 of 28 (8%) logged ≥4 doses per week during this time. Similarly, at month 6 and month 12, 4 of 22 (44%) and 1 of 18 (20%) of those reporting high app acceptability logged ≥4 doses per week via SmartPrEP, respectively.

## Discussion

### Principal Findings

This 12-month feasibility study is one of the first to evaluate an mHealth app designed to support PrEP adherence and HIVST among both cis- and transgender women at elevated risk of HIV acquisition [40]. Despite consistently rating the app as acceptable and receiving quarterly HIV testing and counseling, most participants did not achieve optimal PrEP adherence, demonstrating the limitations of this mHealth app among women at elevated risk of HIV. Most participants did not believe that they were at risk of getting HIV, even though they reported engagement in sexual behaviors associated with PrEP eligibility. Although the study did not formally assess prevention-effective adherence, or the use of PrEP only during periods of risk exposure so that it leads to effective protection against HIV acquisition [41], there was no indication that participants intentionally increased or decreased adherence during periods of fluctuating sexual risk.

We sought to approximate a real-world clinical setting by offering relatively modest compensation for participant time and effort and by limiting study procedures to those included in the CDC’s PrEP clinical guidelines. In contrast to our experience with investigational studies that offer higher compensation, recruitment and retention for this study were challenging. Only 40 participants were enrolled in 19 months, and less than half were retained through the 12-month follow-up period. The difficulty enrolling participants and the low retention illustrate the real-world challenges of PrEP adherence and persistence related to low perceived HIV risk and lack of awareness of HIV prevention tools among women. Historically, lack of awareness of PrEP and insurance coverage challenges have been viewed as barriers to PrEP access among women [42,43], but our results show low PrEP adherence and persistence, in addition to low retention in the study itself, even when insurance coverage and awareness of PrEP were not barriers.

At enrollment, more than half of the participants reported that they did not believe they were at risk of getting HIV. This finding on risk perception was stable throughout the 12-month study period and served as the backdrop to the low adherence and persistence of PrEP use, consistent with the core principles of the health belief model (HBM) [44]. HBM theorizes that individuals who do not personally believe they are susceptible to a disease are not likely to engage in daily preventative behavior, including HIV preventative behavior [45]. While self-perceived risk is influenced by a variety of factors, individuals’ perceived vulnerability to illness is often a necessary precursor for preventative action [46-48]. In this cohort of women, we found that a large majority were engaging in activities that warranted PrEP use; however, only about a third felt that they were at risk of acquiring HIV. This discordance was also reinforced by low HIV prevention awareness, aligning with HBM principles of minimal perceived benefit and ineffective cues to action [44].

Overall, participants found the SmartPrEP app to be moderately to highly acceptable. This was the first time the SmartPrEP was evaluated among a non–men who have sex with men sample, and one of the first mHealth tools assessed among cisgender women [27,28], and almost all participants felt that the app was relevant to them as women. At baseline, participants reported a high likelihood of downloading apps that support their overall health, demonstrating that this was a cohort that wanted to engage with mHealth tools. Across all study visits, most participants scored as acceptable the PrEP reminders and HIVST-related SmartPrEP features. However, there was no correlation between app acceptability and self-reported PrEP adherence via SmartPrEP or use of the HIVST features. As app acceptability and perceived relevance were strong, the low PrEP adherence demonstrated that an app, even when highly acceptable, can only do so much to facilitate engagement in HIV prevention among women.

### Strengths and Limitations

This feasibility study has several strengths. This study was conducted in the Bronx, one of the CDC’s “Ending the HIV Epidemic” high burden counties in the United States [40]. The high burden and potential exposure to HIV were demonstrated by the occurrence of 2 pregnancies, 5 positive STI test results, and 1 HIV seroconversion among 40 participants during the 12-month study period. This study setting, where use of the SmartPrEP app was not directly incentivized, yields results that may apply beyond a clinical trial setting. Additionally, our findings demonstrated the feasibility of collecting real-time self-reported PrEP adherence data via an app, reducing the potential social desirability and recall bias of traditional interviewer-administered data collection methods, while offering enhanced privacy, confidentiality, and safety for historically marginalized groups [22]. This method of data collection greatly reduces costs associated with study site capacity while still yielding an approximate assessment of PrEP use. This study also included qualitative assessments, which will be published separately.

The single-site, nonrandomized study design, however, posed limitations. The small sample size and lack of a control group restricted our ability to conduct detailed quantitative assessments of the true benefit of the app as an mHealth tool to support HIV prevention. The lack of an external adherence measure, such as periodic urine TDF concentrations, limited our ability to ascertain objective levels of effective adherence; however, the SmartPrEP app could track daily adherence, and participants had no incentive to overestimate or underestimate adherence. Instead, the SmartPrEP app likely underestimated true adherence [49] and the actual frequency of test kit use, as some participants may have taken PrEP or used test kits without recording these actions.

### Conclusions

These results provide a greater understanding of the current limitations of mHealth for supporting HIV prevention, and the inability of this app, despite consistent acceptability, to support PrEP adherence without additional interventions. Large-scale public health messaging campaigns, including those focused on PrEP, have been successful in addressing low awareness of HIV and the benefits of HIV prevention [50-53]; however, they have historically lacked a focus on cisgender women [50,51]. Behavioral interventions, including mHealth tools, need to be accompanied by societal-level programs that address gaps in risk perception. The increasing availability of highly effective, long-acting injectable PrEP may not provide sufficient protection for women unless steps are taken to promote PrEP awareness and address the underestimates of HIV risk perception. There is a strong continued need for research on accessible mHealth and other interventions designed to benefit women by promoting PrEP awareness and supporting PrEP.

## Supplementary material

10.2196/86407 Multimedia Appendix 1 Supplementary app information.

10.2196/86407 Multimedia Appendix 2 SmartPrEP app photos.

10.2196/86407 Multimedia Appendix 3 Stratified pre-exposure prophylaxis adherence by sexual behaviors and average app acceptability.

## Abbreviations

CDC: Centers for Disease Control and Prevention
FTC: emtricitabine
HBM: health belief model
HIVST: HIV self-testing
mHealth: mobile health
PrEP: pre-exposure prophylaxis
STI: sexually transmitted infection
TDF: tenofovir disoproxil fumarate
