# Supplementary material for: Pre-Exposure Prophylaxis Adherence and HIV Self-Testing App Among Women in the South Bronx: 12-Month Usability, Acceptability, and Feasibility Study
Source: JMIR Form Res. 2026 Jun 2;10:e86407. doi: 10.2196/86407 (PMC13229397; doi:10.2196/86407)
Supplement: Multimedia Appendix 1 [file formative-v10-e86407-s001.docx]

*Previous SMARTtest app*

The development of the current SmartPrEP app was based on the “SMARTtest” app, previously developed by Sia et al. through a user-informed process to support HIV and syphilis self-testing for MSM and transgender women in New York City [1].

*SmartPrEP App overview*

The SmartPrEP app was built using the ReactNative framework and included: 1) private user account creation; 2) detailed instructions on how to perform a rapid OraQuick HIV test on oneself or partners; 3) objective and automated test scanning and results; 4) the capacity to share, delete, or save results; and 5) information on additional health resources and nearby clinics. A machine-learning algorithm was integrated into the app for a more objective interpretation of test results, using image recognition and processing tailored to HIV rapid test images.

Study participants were instructed at enrollment and reminded at follow-up visits that any positive self-test result was not diagnostic and that a repeat test must be conducted at the site. Of note, the use of the app was not required to see the OraQuick self-test results. Instead, the app was designed to help participants, if they opted to do so, to interpret and log the results of the FDA-approved OraQuick self-testing kit.

*App front end*

Study staff assisted participants in downloading the "SmartPrEP" smartphone app and with the initial login (Supplemental Figure 1: Panel A) and user authentication. Each participant then had the option to view an instructional video on how to use the HIVST kit and go through the step-by-step instructions for performing an HIVST. Following these testing instructions, the participant was prompted to choose the testing mode (panel C) and take a picture of the test device with the help of an assistive-imaging-grid to ensure that the phone’s camera was oriented parallel to the kit. The image was sent to an internet server hosted by Amazon Web Services (AWS) with the trained machine learning classification model for automated positive or negative classification. The user then received the determination, and had the option to save, share or delete the results. The resources tab (Supplemental Figure 1: Panel B) gave the participant the option to access a calendar to monitor their PrEP adherence, access more information about the test kit (as provided by the manufacturer), navigate to CDC resources on HIV, dial a CDC hotline number for immediate assistance or counseling, and search for nearby clinics.

*App back end*

A commercial cloud database service was used to securely store account creation metadata; date, time, results and unique ID of each conducted HIVST. Participants were able to see these details about their HIVST. Third-party application of programming interfaces (APIs) allowed participants to share results via text and email messaging with third parties of their choosing.

*Adherence support*

The SmartPrEP app incorporated a daily PrEP adherence monitoring feature where participants entered confirmation that they took their dose into the app (panel G). Adherence reminders were facilitated by push notifications sent prior to the time of the scheduled dose or only when the participant was late to enter a confirmation that they took the scheduled dose. User preferences for these notifications were configured at the time of app installation.

*HIVST/Partner Testing guidance*

Participants could select the testing options displayed on the ‘HIV Testing Options’ page. The ‘Just Me’ and ‘Duo Test’ options both offered the option to save and share results with clinicians, family members and/or partners. The ‘Duo Test’ had the additional option to test a partner and not save the results. The ‘Just My Partner’ option gave the user the ability to test their partner without saving or sharing the results. For this option, a visual prompt would appear on the app requesting the partner to consent or decline to upload their HIV self-test results to the study’s database.

*SmartPrEP Image processing using machine learning:*

The image processing component was broken down into two sections: preprocessing, and results prediction. The photographic image of the HIV test kit used by the participant was sent to a preprocessing server where the test kit’s boundary was detected, and the image cropped. The cropped image was then sent to the deep learning-based prediction layer for interpretation of the test result.

*SmartPrEP APP HIV Self-test/Partner Test Communique to Participants:*

Participants were provided reinforced counseling detailing privacy features of the app by study staff. Prior to and after the app installation, participants were reminded that there were only unique identifiers associated with each self-test and partner test, with password-protected access and that no information that could identify the participant was stored in the cloud. Moreover, participants were reminded that HIV self-/partner test results would only be stored in the cloud database and not on their phone.

*SmartPrEP Guidance:*

Upon installation, the study staff provided app education for correct use. Additionally, to assist the participant in installing the SmartPrEP app on their mobile phone, the study staff educated the participant on app features and functions, such as setting alerts and reminders regarding daily PrEP intake. Study staff also provided a tutorial on how to use the HIV self-test and/or partner test features.

**References**

1. Rael CT, Kutner BA, Lentz C, Lopez-Ríos J, Dolezal C, Arumugam S, Sia S, Balán IC. Transgender women’s experiences using SMARTtest, a smartphone application to facilitate self- and partner- HIV/syphilis testing using the INSTI Multiplex: A brief report. Arch Sex Behav 2023 Jul;52(5):1961–1968. PMID:37188893
