# Supplementary material for: Pre-Exposure Prophylaxis Adherence and HIV Self-Testing App Among Women in the South Bronx: 12-Month Usability, Acceptability, and Feasibility Study
Source: JMIR Form Res. 2026 Jun 2;10:e86407. doi: 10.2196/86407 (PMC13229397; doi:10.2196/86407)
Supplement: Multimedia Appendix 2 [file formative-v10-e86407-s002.docx]

**
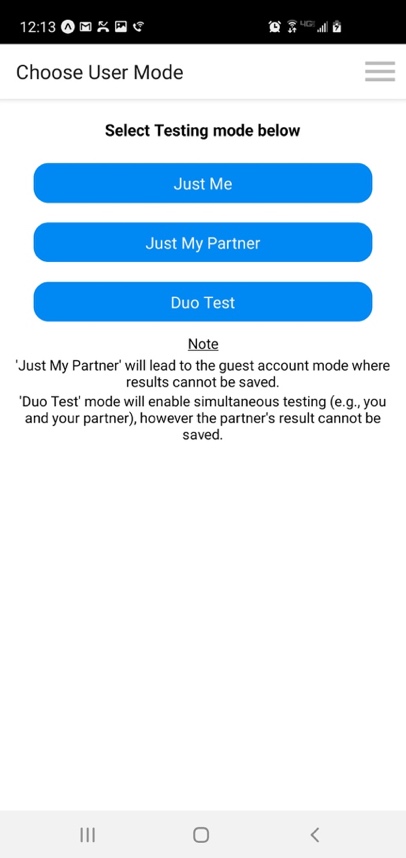

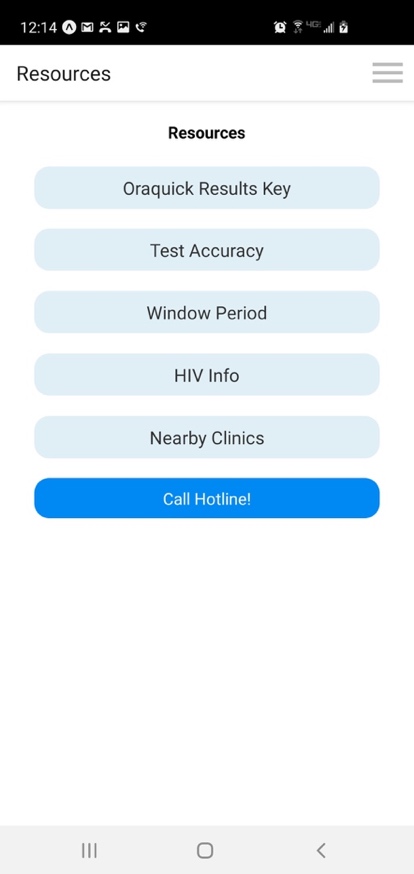

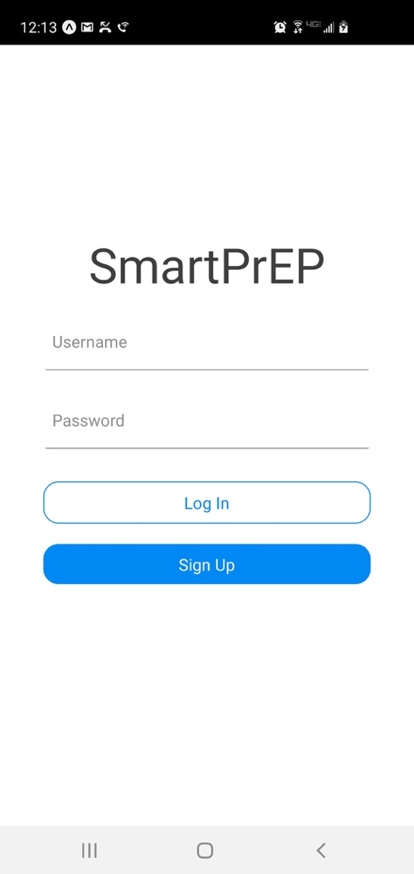
SmartPrEP App Photos**

**C. HIV Testing Options**

**B. Resource Page**

1. **User Log-In**

**
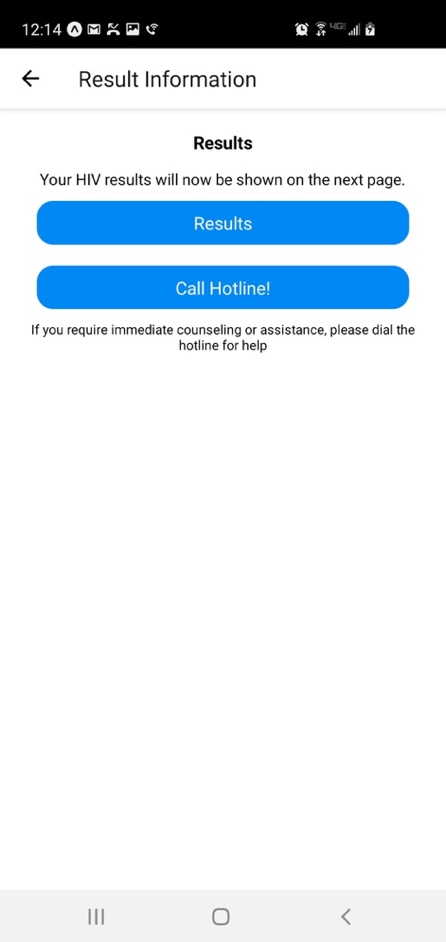
**

**
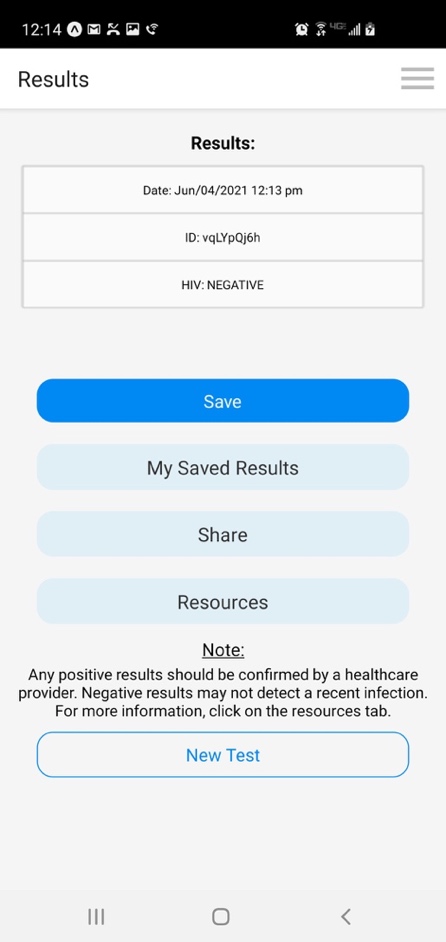

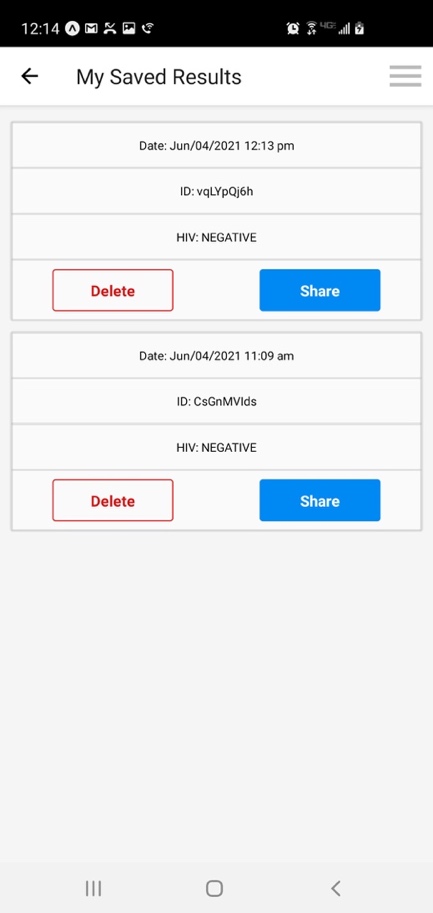
**

**F. HIV Test Result Log**

**E. HIV Test Result Page**

**D. HIV Test Result Landing Page**

**
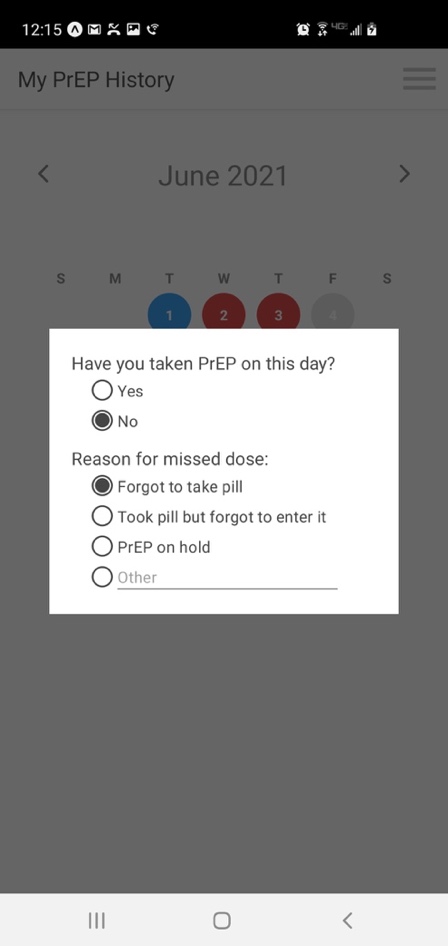

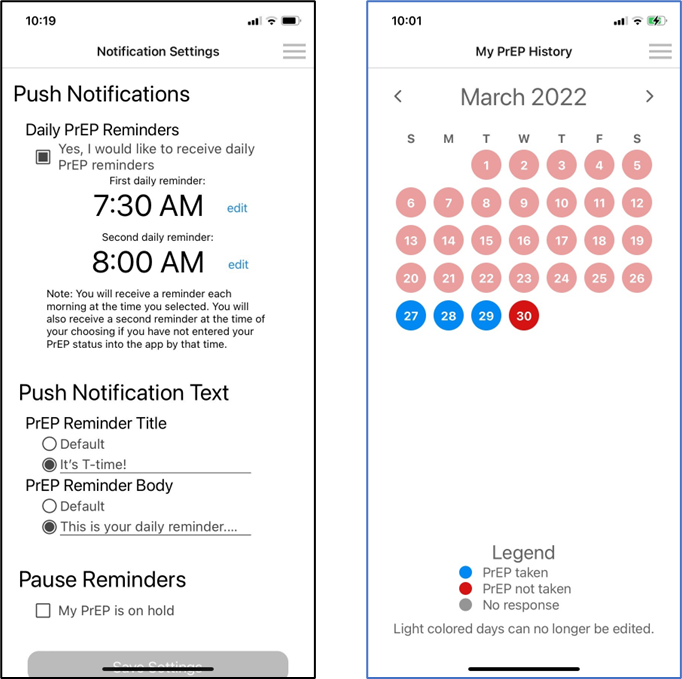
**

**
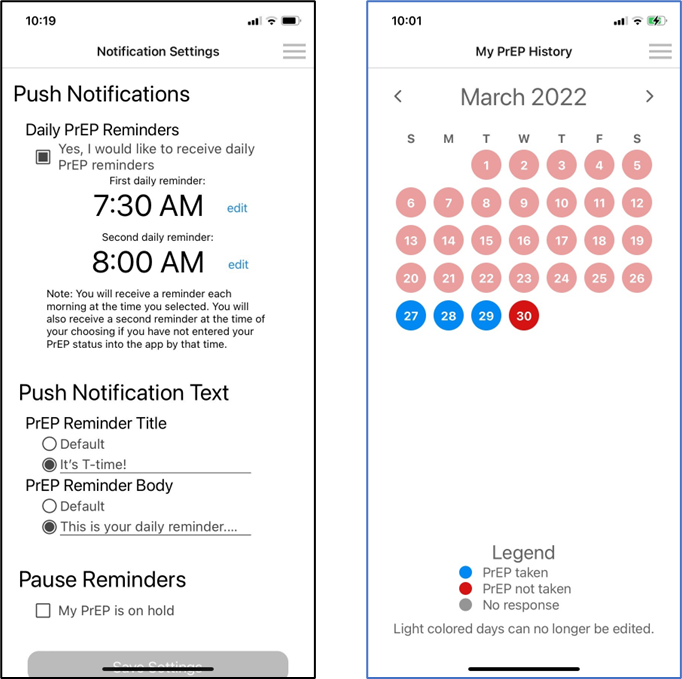
**

1. **PrEP History Response and Log**

**H. PrEP History Calendar**

**G. PrEP Notification Reminder Settings**
