# Supplementary material for: Pre-Exposure Prophylaxis Adherence and HIV Self-Testing App Among Women in the South Bronx: 12-Month Usability, Acceptability, and Feasibility Study
Source: JMIR Form Res. 2026 Jun 2;10:e86407. doi: 10.2196/86407 (PMC13229397; doi:10.2196/86407)
Supplement: Multimedia Appendix 3 [file formative-v10-e86407-s003.pdf]

**Stratified PrEP Adherence**	**Month 3 (N=28)**		**Month 6 (N=22)**		**Month 12 (N=18)**	
Question	N	%	N	%	N	%
**Responded "Yes" to ≥ 1 Sexual Behavior Associated with HIV acquisition in the past 3 months**	**Optimal[a]**	**Sub-Optimal[a]**	**Optimal[a]**	**Sub-Optimal[a]**	**Optimal[a]**	**Sub-Optimal[a]**
Yes	3 (18%)	14 (82%)	5 (42%)	7 (58%)	2 (22%)	7 (78%)
No	2 (18%)	9 (82%)	0 (0%)	9 (100%)	1 (11%)	8 (89%)
**Average App Acceptability**	**Optimal[a]**	**Sub-Optimal[a]**	**Optimal[a]**	**Sub-Optimal[a]**	**Optimal[a]**	**Sub-Optimal[a]**
High (4-5)	1 (8%)	11 (92%)	4 (44%)	5 (56%)	1 (20%)	4 (80%)
Moderate (2-4)	4 (25%)	12 (75%)	1 (8%)	11 (92%)	2 (15%)	11 (85%)
Low (1-2)	0 (0%)	0 (0%)	0 (0%)	0 (0%)	0 (0%)	0 (0%)

[a] *Optimal PrEP adherence defined as >= 4 weekly doses self-reported via SmartPrEP*
